# External quality assessment for CD4 + T-lymphocyte count test: Performance of the Brazilian public health laboratories network

**DOI:** 10.1097/MD.0000000000010125

**Published:** 2018-05-25

**Authors:** Pâmela Cristina Gaspar, Bruna Lovizutto Protti Wohlke, Milena Karina Coló Brunialti, Ana Flávia Pires, Igor Massaki Kohiyama, Reinaldo Salomão, José Boullosa Alonso Neto, Orlando da Costa Ferreira Júnior, Miriam Franchini, Maria Luiza Bazzo, Adele Schwartz Benzaken

**Affiliations:** a Department of Surveillance, Prevention and Control of Sexually Transmitted Infections, HIV/AIDS and Viral Hepatitis (DIAHV), Secretariat of Health Surveillance, Ministry of Health, Brasília, Distrito Federal; b Immunology Laboratory/Division of Infectious Diseases/Escola Paulista de Medicina/Federal University of São Paulo, São Paulo; c Laboratory of Molecular Virology, Institute of Biology, Federal University of Rio de Janeiro, Rio de Janeiro, Rio de Janeiro; d Laboratory of Molecular Biology, Microbiology and Serology, Department of Clinical Analysis, Health Sciences Center, Federal University of Santa Catarina, Florianópolis, Santa Catarina, Brazil.

**Keywords:** CD4+ T-lymphocyte, external quality assessment, quality, test performance

## Abstract

The National Network for CD4+ T-lymphocyte counting of Brazil comprises 93 laboratories. This study reports the laboratory performances achieved in external quality assessment (EQA) rounds provides by Ministry of Health to evaluate the quality of the kits used and the performance of test by the technicians.

Ten EQA rounds were analyzed according the EQA criteria aimed to evaluate individual laboratory performance on the basis of the accuracy of their results compared to the general mean obtained by all participating laboratories and the reproducibility of the results obtained between 2 samples from the same donor.

The percentage of approved and failed laboratories in the EQAs tends to follow a uniform pattern. Since 2011, approval has remained above 80% and the failure rate has never exceeded 15%.

EQA is very important to evaluate the performance of the laboratories, to identify monitor, and to resolve errors as quickly as possible.

## 1 Introduction

Diagnosis and monitoring of HIV infection in Brazil is offered free of charge for all users of the National Unified Health System (Sistema Único de Saúde – SUS in Portuguese). The laboratories responsible for these services are organized in a national networks financed by the Ministry of Health (MoH).^[1]^ These include the National Network for CD4+ T-lymphocyte Counting. Created in 1997 with an initial 32 laboratories,^[2]^ the network currently comprises 93 laboratories throughout Brazil ensuring that the entire Brazilian population has access to the testing facilities.

**Figure 1:**
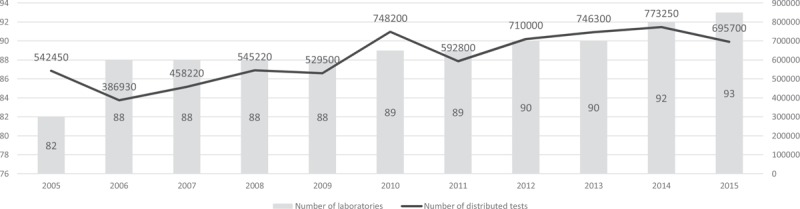
Graph showing the increasing number of laboratories belonging to the National Network for CD4 + T-lymphocyte Counting over the years, associated to the increasing number of tests distributed per year between 2005 and 2015 by the Ministry of Health.

In 1997 to 2001, the MoH provided 558,700 CD4/CD8 tests to all Brazil's states and introduced measures to expand the installed capacity and technical quality of the national laboratories network and reduce waiting times for results. Subsequently, in 2002 to 2004, through the ministerial directive SAS/MS no 172, published in 2001,^[3]^ the federal government made individual states responsible for the network, but this brief decentralization experience was not successful. Reports of shortages of reagents, long waiting times to access tests, and a series of other problems convinced the MOH to recentralize the purchase, distribution, and quality control of the CD4 counting in the entire country. This was done in 2004, through the ministerial directive no 1.015,^[4]^ and in the following 10-year period the MoH more than doubled the number of tests for TCD4/CD8 lymphocyte counting (from 631,512 in 2005 to 1,300,000 in 2015) and increased the number of laboratories in the network by some 16% (Fig. 1). The MoH also succeeded in negotiating lower prices for tests (per unit) – from US$15.08 (exchange rate: 2.5) in 2005 to US$7.36 (exchange rate: 3.55) in 2015, producing an overall saving of 51.19% despite the unfavorable US$ exchange rate in local currency.^[5]^

The equipment, calibrators, and the kits used for the procedure in Brazil are procured through an annual public bidding process, with a public tendering notice carefully detailing the technical specifications, supplies, and accessories required.^[6,7]^ The number of kits needed is based on HIV epidemiological data and other criteria governed by current Clinical Protocols and Therapeutic Guidelines.^[8]^ The winning bidder is submitted to a “trial phase” in which it must provide one complete kit for performing the test in order to verify that the product meets the requirements of the public notice. Contracts are signed definitively only when this “trial phase” is successfully completed and only when the purchasing party is totally satisfied that the winning suppliers can guarantee “full service” (ie, kits, ancillary equipment).

Absolute and relative numbers of CD4/CD8 T lymphocytes are determined by flow cytometry. Currently, the tests are performed with the BD FACSCalibur BD equipment using BD Multitest CD3 FITC/CD8 PE/CD45 PerCP/CD4 APC, BD TruCOUNT^TM^ Absolute Count Tubes and BD FACS^TM^ Lysing Solution. In addition, it was used BD TruCONT Control Beads, BD CALIBRITE^TM^ 3 Beads and BD CALIBRITE APC and also all the inputs to ensure perfect test performance.

In 1996, the MoH introduced the National Program for Quality Assurance of Laboratory Tests – external quality assessment (EQA) to evaluate the quality of the kits used and the performance of test by the technicians.^[9]^ Three EQA rounds are done per year by sending panels to the 93 laboratories. In the event of a laboratory failing to perform satisfactorily, the company supplying the kits is obliged to conduct a technical visit to apply corrective measures, including retraining the laboratory staff to resolve any issues that might affect the quality of testing. It is important to emphasize that the supplier company is totally responsible for this (including staff training) in strict accordance with the contract conditions. A total of 20 EQAs had been performed by year 2015.

The first EQA was conducted in 1999 through a technical cooperation agreement with the University of California at Los Angeles. Aimed at assisting the implementation of the Brazilian program, this involved University of California at Los Angeles providing 2 shipments of samples from Canada's external assessment program, coordinated by the National Laboratory for Analytical Cytology in Ottawa.^[10]^ During the following year a partnership set up between the MOH and with the Immunology Laboratory of the Division of Infectious Diseases from Escola Paulista de Medicina (EPM)/Universidade Federal de São Paulo led to the development of a 3-stage pilot project to provide technical support for implementing CD4 EQA using fresh blood samples collected in EDTA.

Since 2011, the EQAs of CD4/CD8 T lymphocyte counting consist of a panel produced by the Immunology Laboratory of the Division of Infectious Diseases – EPM/Federal University of São Paulo), costing 50% less than the “market price” of the international panels. The panels are composed of peripheral blood samples containing different numbers of CD4/CD8 T lymphocytes, and one of the samples was sent in duplicate to the laboratory. The laboratories receive the panel, unaware of the number of cells in the samples, and perform routine lymphocyte counting. The results are then reported on the Qualilab online system (qualilab.aids.gov.br). The laboratories are identified by a randomly selected code designed to protect their original identification. This procedure ensures the confidentiality of the results and the complete lack of bias on the analysis of the data produced in each laboratory thus ensuring the impartiality of the Advisory Committee members.

Using the results sent by the participating laboratories, the EQA evaluates the accuracy of the results by comparing the general mean and standard deviations (SDs), as well as the reproducibility, by analyzing the results obtained from the 2 duplicate samples contained in the panel which were obtained from the same donor. Analysis of the EQAs involves a scoring system to rate the performance of the participating laboratories in terms of “excellent,” “approved,” or “failed.” This study reports the laboratory performances achieved in EQAs rounds 11 to 20, and confirms the importance of the EQA programs to monitor current errors and evaluate the performance of the 93 laboratories.

## 2 Materials and methods

Ten EQAs were analyzed (EQA11–20). These assessments were carried out between 2011 and 2015. In EQA 11, 85 laboratories were analyzed, 82 in EQA 12, 88 in EQA 13, 88 in EQA 14, 86 in EQA 15, 84 in EQA 16, 88 in EQA 17, 87 in the EQA 18, 86 in EQA 19, and 91 in EQA 20. From the results entered in the Qualilab system by the laboratories it was possible to obtain the mean and SDs of the lymphocyte counts of the samples. The scoring criteria used in the evaluations of the participating laboratories are set out in Table 1. These criteria aimed to evaluate individual laboratory performance on the basis of the accuracy of their results compared to the general mean obtained by all participating laboratories and the reproducibility of the results obtained between 2 samples from the same donor. The maximum possible score in the overall analysis of the 4 samples was 64 points, considering the counts of the CD45, CD3, CD4, and CD8 receptors. The reproducibility analysis makes it possible to score up to 16 points. According to the sum of the scores obtained in the 2 analyzes (which could be a maximum of 80 points) the laboratories were rated as described in Table 2.

**Table 1:**
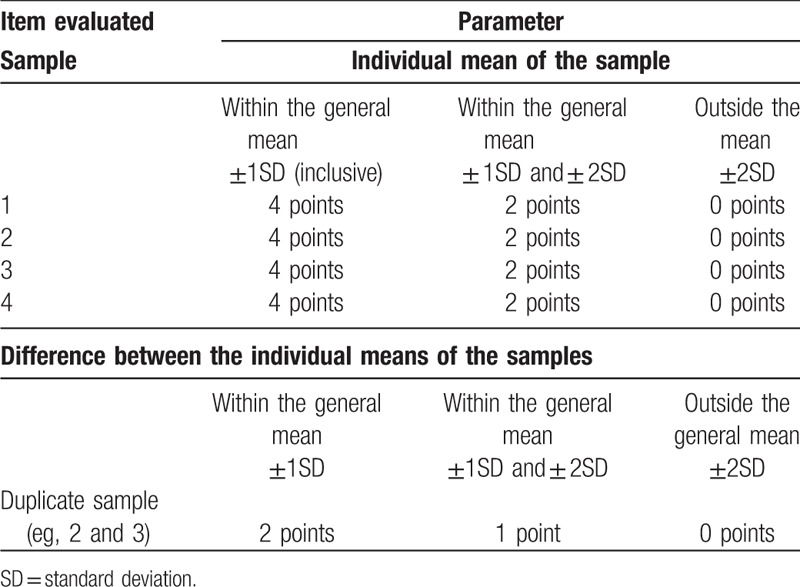
Scoring system according to the parameters analyzed in external quality assessment for CD4 + T-lymphocyte count test.

**Table 2:**
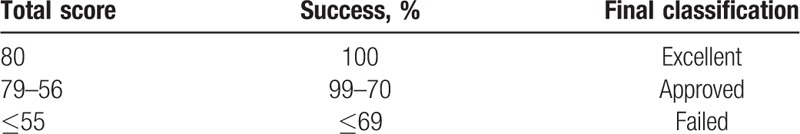
Classification of the laboratories according to the score obtained and the percentage of success.

Ethical approval was not necessary because the data collected is not personal identifiable.

### 2.1 Samples used

Samples of peripheral blood collected in a vacuum tube containing K2 EDTA (BD Franklin Lakes, NJ) from 3 different donors were collected at the Immunology Laboratory of the Division of Infectious Diseases – EPM/Federal University of São Paulo.

After homogenization, the samples were divided into aliquots with an approximate volume of 300 μm and stored in microtubes, sufficient to form panels with 4 samples each. The panels were packed in a container to prevent tipping of the samples during shipping. They were then packaged in polystyrene boxes containing two 500 mL recyclable ice sheets previously refrigerated at 2 to 8 °C in order to minimize possible panel temperature variation during transport. On the same day the panels were sent to all the laboratories of the Network. Each panel transport box contained “Instructions for using the panel for the EQA of CD4+/CD8 + T-lymphocyte counts   EQA,” and guidelines for laboratories to perform the tests. The results were entered by the laboratory personnel in the Qualilab system and analyzed using the Microsoft Excel program (Microsoft Corporation, Seattle, WA).

### 2.2 Analysis conducted

#### 2.2.1 Overall analysis (4 samples)

Based on the results released in the Qualilab system by each EQA participant, the mean and SDs were calculated for each of the laboratories analyzed in the different samples, with the addition or subtraction of 1 or 2 SDs. Each analyzed variable was scored according to the range into which the result fell. The maximum possible score in this analysis was 64 points (Table 1).

#### 2.2.2 Reproducibility analysis

A new analysis of the samples collected from the same donor was made for the reproducibility analysis. The results generated by these samples were analyzed as if they were only from one sample, for example, they generated a single mean and an SD for each one of the variables measured. The maximum possible score in the reproducibility analysis was 16 points (Table 1).

### 2.3 Final score of the laboratories

From the sum of the points received by the laboratories in the global and reproducibility analyses the percentage of success was calculated based on the score of each laboratory. The laboratories scoring 100% were rated “excellent,” those with 99% to 70% were “approved” and those with ≤69% were failed (Table 2).

## 3 Results

Table 3  shows the results of each participating laboratory in the 10 evaluations carried out between 2011 and 2015. In terms of “failures” (ie, percentage of laboratories with ≤69% of successes or those with scores of ≤55, in the EQA 11, 10.6% of the participating laboratories were failed, 14.6% in EQA 12, 10.2% in EQA 13, 9% in EQA 14, 12.8% in EQA 15, 7.0% in EQA 16, 13.6% in EQA 17, 10.3% in EQA 18, 15.0% in EQA 19, and 7.7% in EQA 20.

**Table 3:**
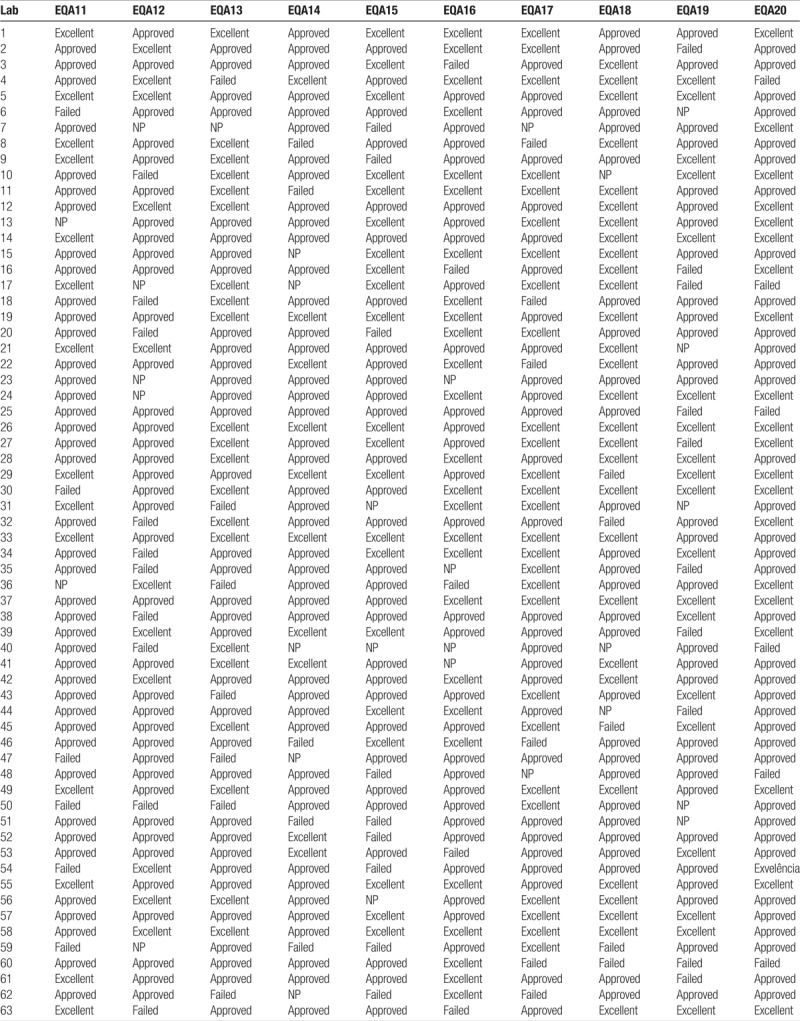
Results of the external quality assessments in the CD4 network (EQA11–20).

**Table 3 (Continued):**
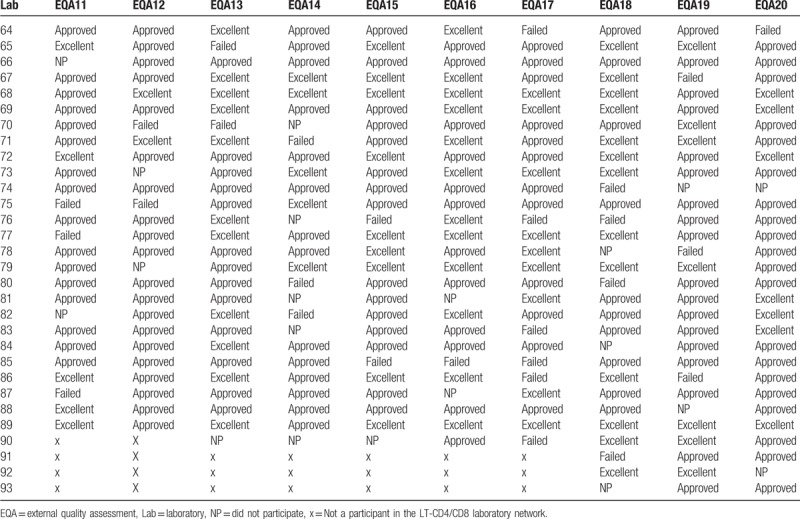
Results of the external quality assessments in the CD4 network (EQA11–20).

Regarding the participation of the laboratories in each assessment, 95.5% participated in EQA 11, 92.1% in EQA 12, 97.7% in EQA 13, 88.8% in EQA 14, 95.6% in EQA 15, 93.3% in EQA 16, 97.8% in EQA 17, 93.5% in EQA 18, 92.5% in EQA 19, and 97.9% in EQA 20.

**Figure 2:**
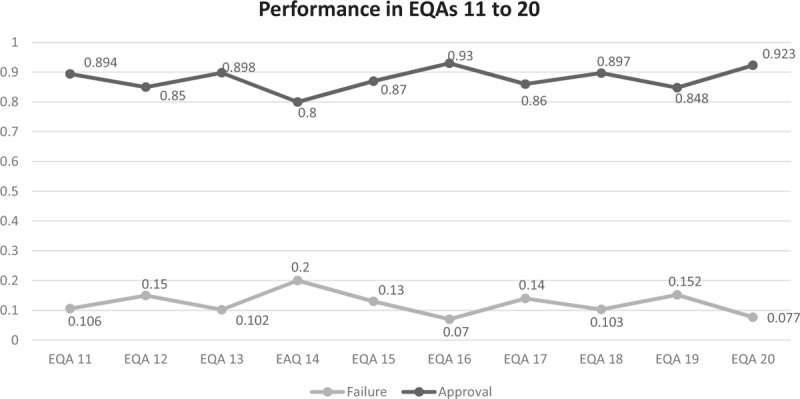
Graph showing the uniform pattern of the percentage of the laboratories approved and disapproved per round between 11 and 20 external quality assessment for CD4 + T-lymphocyte count test rounds.

A percentage of 89.4 participating laboratories were awarded excellent/approved status in EQA 11, 85.0% in EQA 12, 89.8% in EQA 13, 80.0% in EQA 14, 87.0% in EQA 15, 93.0% in EQA 16, 86.0% in EQA 17, 89.7% in EQA 18, 84.8% in EQA 19, and 92.3% in EQA 20. Figure 2 shows the performance of laboratories in EQAs (EQA11–20) rated “approved” and “failed.” It can be noted that the percentage of approved and failed laboratories in the EQAs tend to follow a uniform pattern. Since 2011, approval has remained above 80% and the failure rate has never exceeded 15%.

Analysis of Table 3  reveals the identity of the laboratories with recurrent failures or low EQA participation. The laboratories that presented 3 or more situations that were not positive (ie, failed or did not participate) were selected for evaluation. Using these parameters, we identified 15 laboratories (of the total of 93) of which 12 were rated as “approved” interspersed with failures or nonparticipation. Three laboratories were considered more critical, since they presented consecutive failures and which had failed to participate consecutively in the last EQA round. This analysis enabled more specific strategies and actions to be structured for the most critical laboratories, with the possibility of carrying out more frequent follow-ups and retraining the professional staff.

## 4 Discussion and conclusions

This study describes the methodology used to evaluate the laboratories that comprise the CD4/CD8 T lymphocyte counting network of the Brazilian MoH. The evaluations respond to a need to ensure reliable results to the users of the National Unified Health System.

All the laboratory procedures were performed using the FACSCalibur 4-color equipment from the BD Bioscienses company, and the set of specific immunological reagents required for identifying cell surface markers, using flow cytometry, containing CD3/CD4/CD8/CD45 monoclonal antibodies. Other reagents such as controls, calibrators, and solutions were also provided by the supplier, as established in the contract, were also used in order to pursue the activities related to laboratory monitoring of the immunological defenses of HIV-infected patients, performing analyzes of peripheral blood samples for counting T lymphocytes and their subpopulations.

A comprehensive report is drawn up at the end of each evaluation process, containing information about the panel, its preparation, evaluation criteria, and the results. This is made available to all the laboratories in the Network and published on the MoH website. The confidentiality of the participants is respected. This methodology made it possible to evaluate and classify the laboratories belonging to the CD4/CD8 T lymphocyte network. Following the evaluation process all laboratories that perform satisfactorily receive a certificate of excellence or approval, according to their scores. Failed laboratories receive a technical visit from the company responsible for supplying the equipment and supplies. The company then issues a report of the visit, the operational, infrastructural, or equipment-related problems encountered, and provides other relevant information for the MoH to take corrective action to improve the quality of the laboratory network and the testing services for patients.

EQA for CD4 + T-lymphocyte count test is also established in several countries for more than 30 years, like the “The Quality Assessment and Standardization for Immunological Measures Relevant to HIV/AIDS” since 1997. The quality assessments worldwide also analyze interlaboratory variation like Brazil in order to obtain uniform results. Regarding sample type, the quality assessment programs usually use stabilized blood samples while in Brazil the samples are not stabilized. The blood samples are collected and shipped to the laboratories on the same day and the panels should arrive within 48 hours. Another difference is that Brazilian National Network for CD4 + T-lymphocyte Counting has the same equipment, calibrators, and the kits used for the procedure for all laboratories. When laboratories fail at the quality assessment, for all the experiences found, they also have specific actions to improve the quality as in Brazil.^[10–12]^

EQA contributes to reduce interlaboratory variation and also immunophenotyping error by promoting re-education of the technicians, routine adjustments, and equipment maintenance when necessary. In conclusion, EQA is very important to evaluate the performance of the laboratories, and to identify, monitor and resolve errors as quickly as possible.

## Acknowledgments

The authors thank the Department of Surveillance, Prevention and Control of Sexually Transmitted Infections, HIV/AIDS and Viral Hepatitis, of the Secretariat for Health Surveillance of the Ministry of Health for providing the data of this study.

## Author contributions

**Conceptualization:** A.S. Benzaken, A.F. Pires, I.M. Kohiyama, M.K.C. Brunialti, O.d.C.F. Júnior, P.C. Gaspar, R. Salomão.

**Data curation:** M.K.C. Brunialti.

**Formal analysis:** A.F. Pires, M.K.C. Brunialti.

**Funding acquisition:** A.S. Benzaken, A.F. Pires, J.B. Alonso Neto, M.L. Bazzo, M. Franchini, P.C. Gaspar.

**Investigation:** A.S. Benzaken, B.L.P. Wohlke, M.K.C. Brunialti, M. Franchini, O.d.C.F. Júnior, R. Salomão.

**Methodology:** B.L.P. Wohlke, M.L. Bazzo, M.K.C. Brunialti, R. Salomão.

**Project administration:** A.S. Benzaken, A.F. Pires, B.L.P. Wohlke, I.M. Kohiyama, J.B. Alonso Neto, M.L. Bazzo, M.K.C. Brunialti, M. Franchini, O.d.C.F. Júnior, P.C. Gaspar.

**Resources:** B.L.P. Wohlke, M.K.C. Brunialti.

**Supervision:** B.L.P. Wohlke.

**Validation:** M.K.C. Brunialti.

**Writing – original draft:** B.L.P. Wohlke, I.M. Kohiyama, M.K.C. Brunialti, P.C. Gaspar.

**Writing – review & editing:** A.S. Benzaken, A.F. Pires, B.L.P. Wohlke, I.M. Kohiyama, J.B. Alonso Neto, M.L. Bazzo, M.K.C. Brunialti, M. Franchini, O.d.C.F. Júnior, P.C. Gaspar, R. Salomão.
